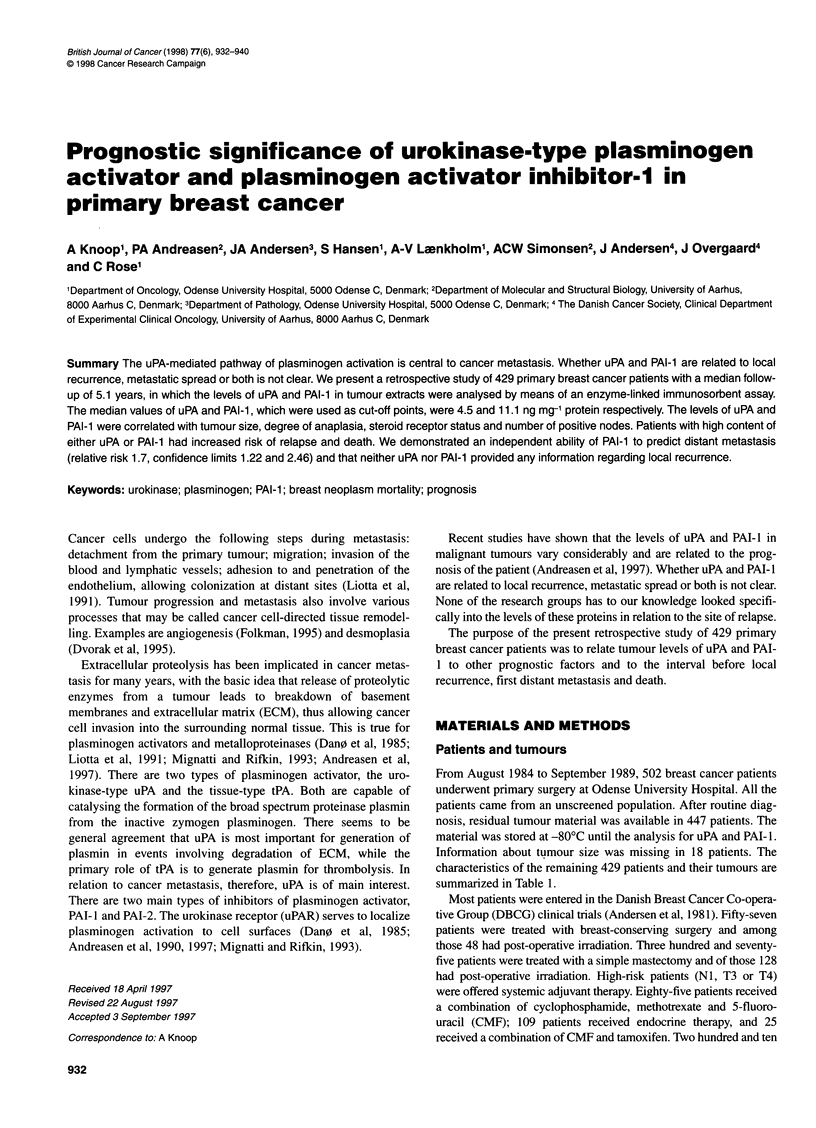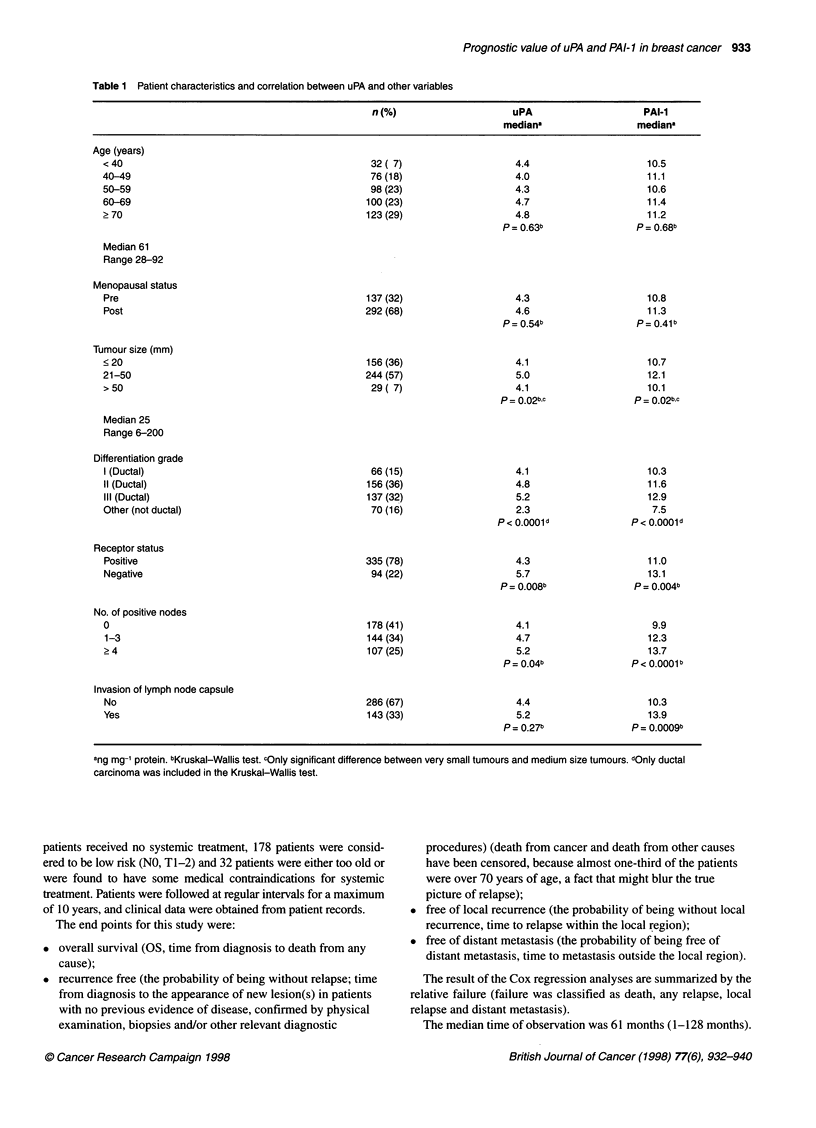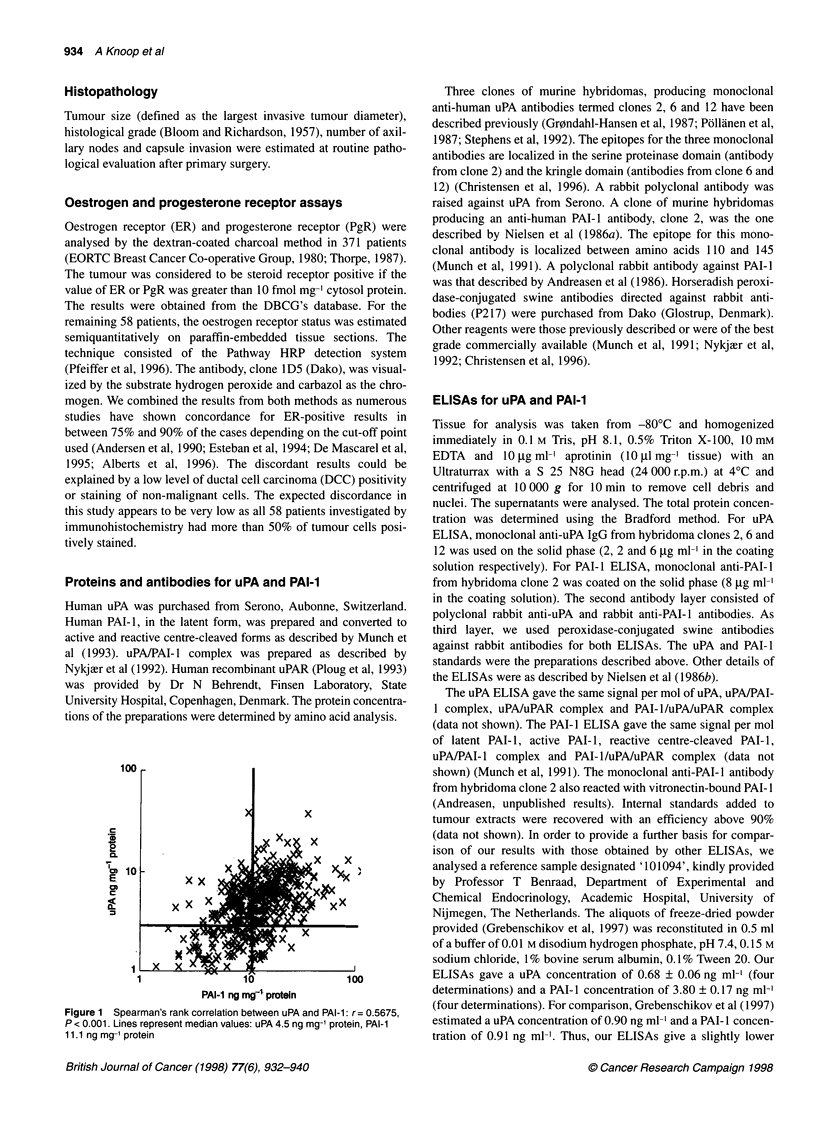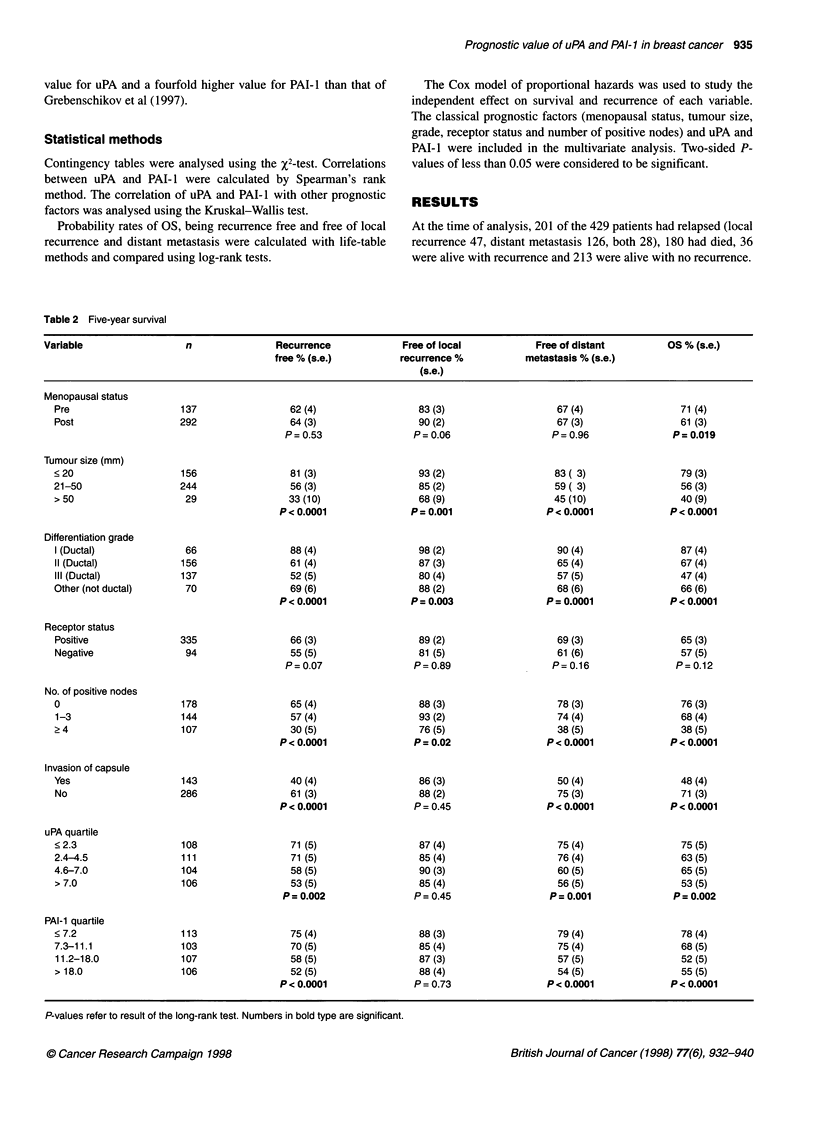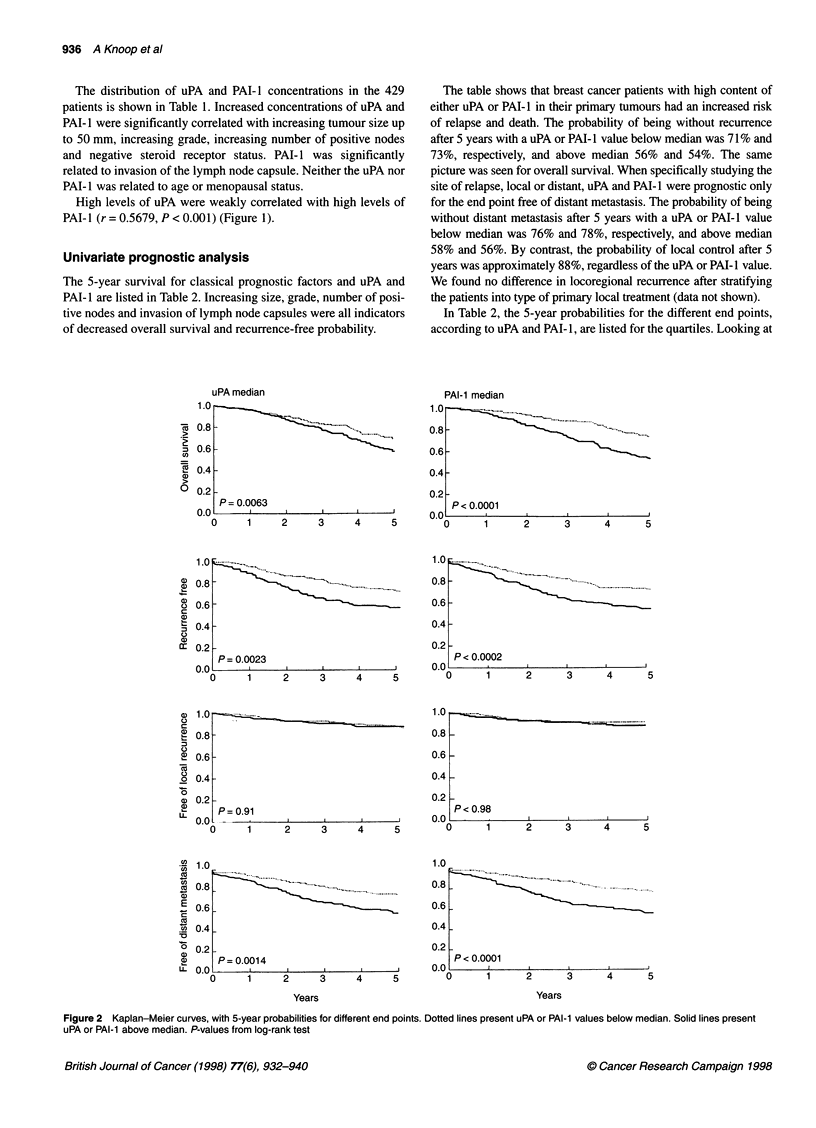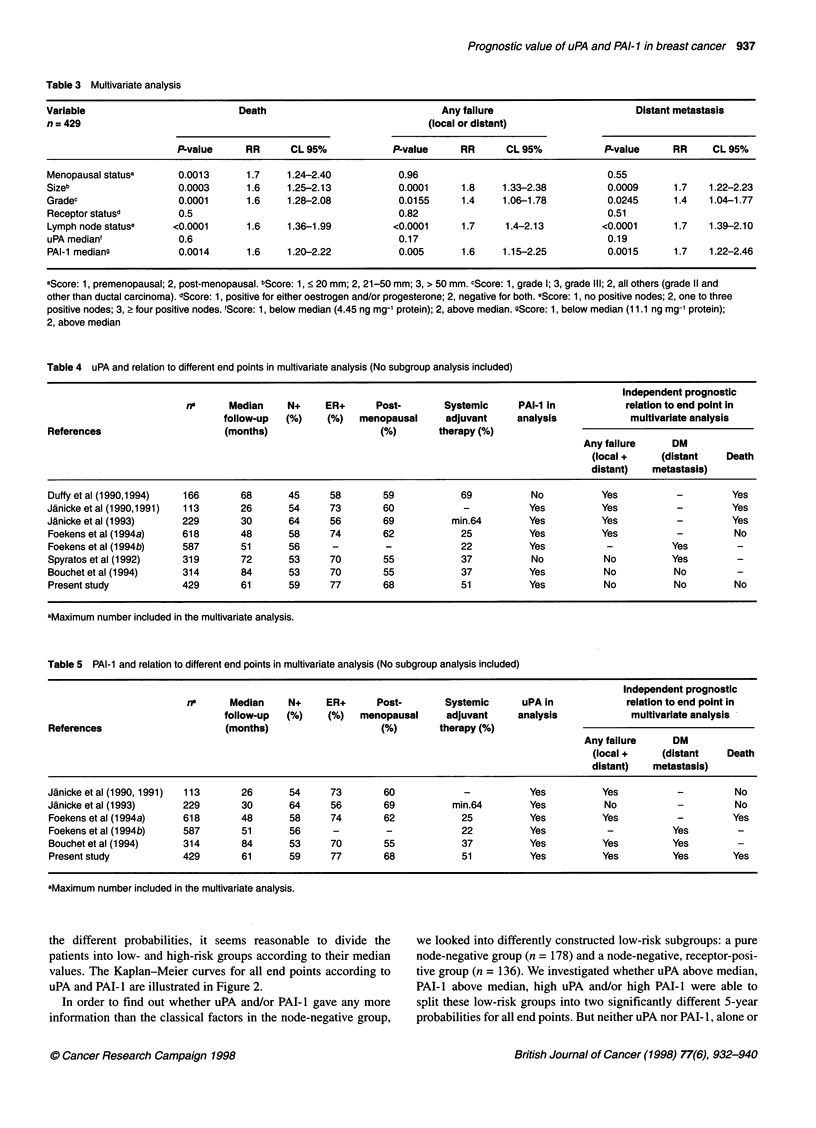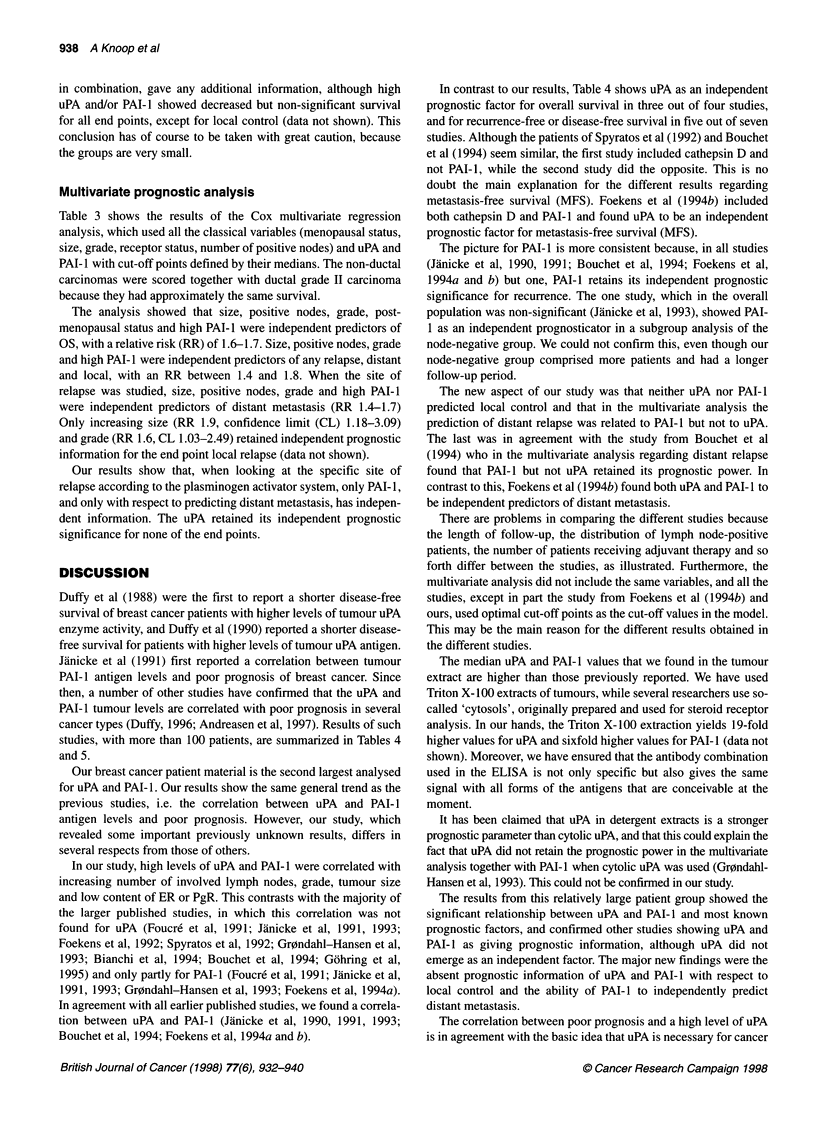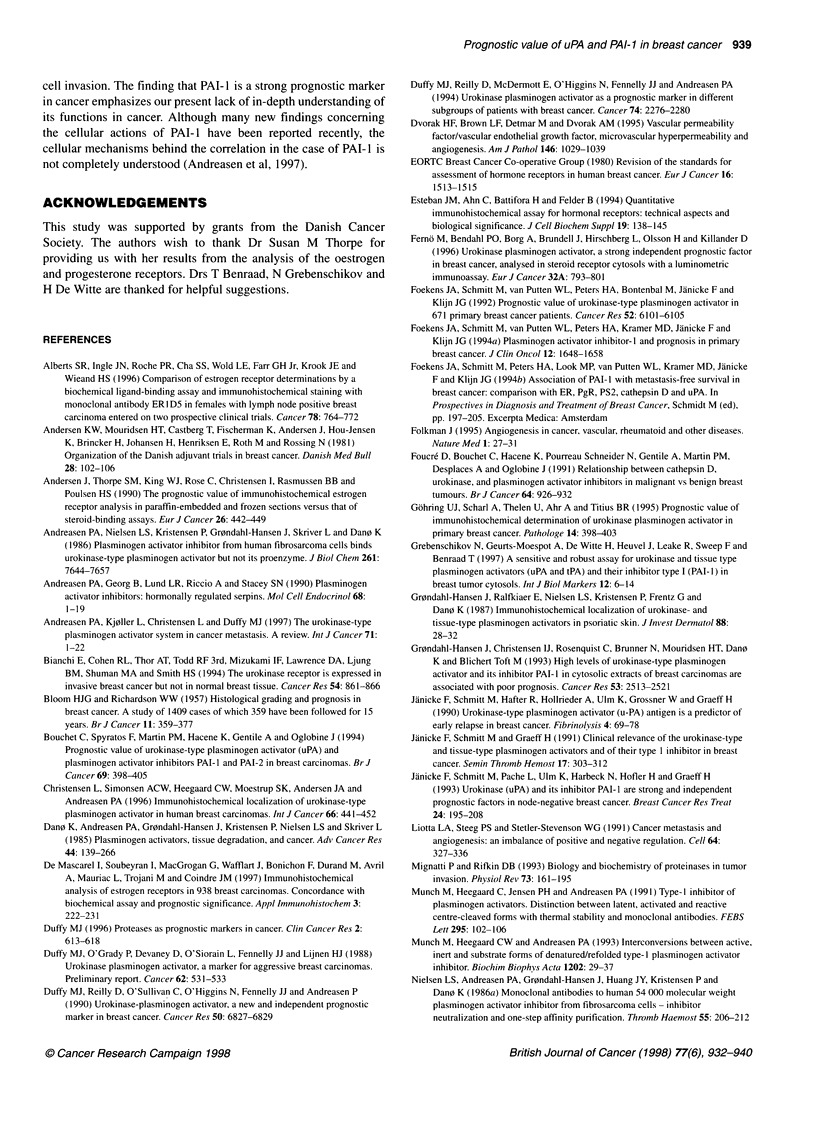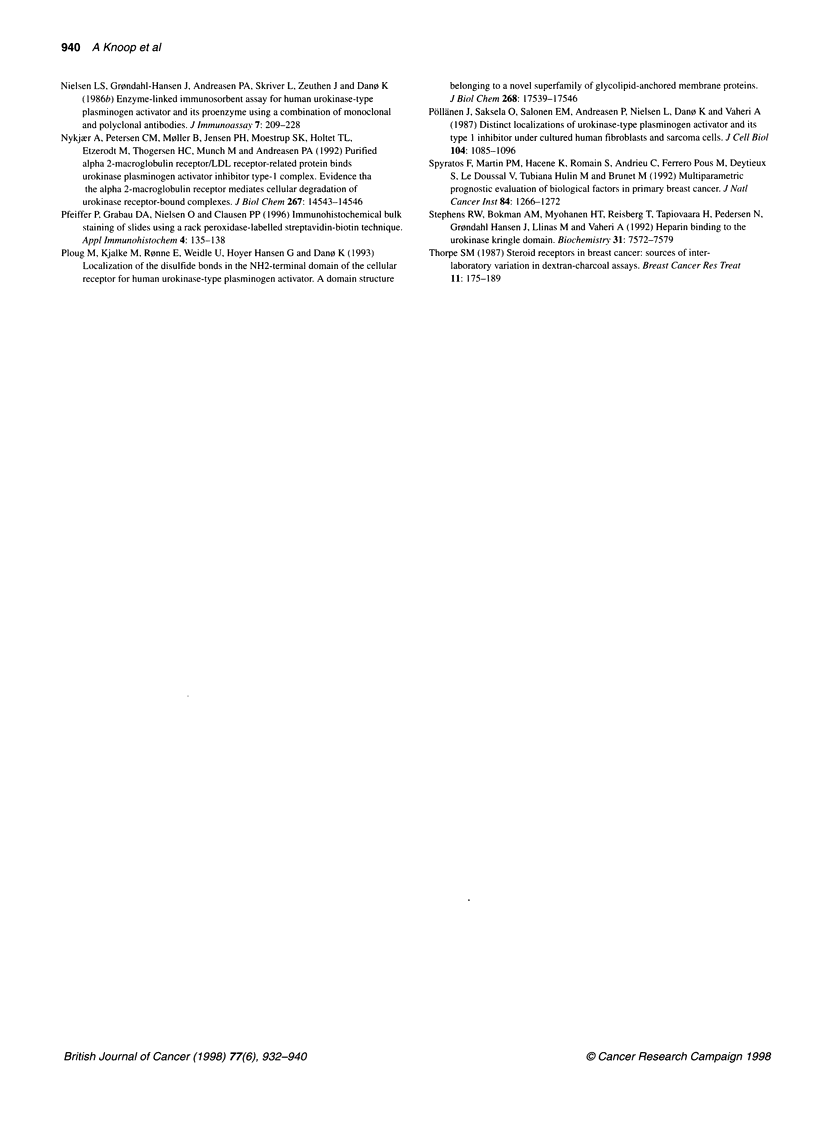# Prognostic significance of urokinase-type plasminogen activator and plasminogen activator inhibitor-1 in primary breast cancer.

**DOI:** 10.1038/bjc.1998.154

**Published:** 1998-03

**Authors:** A. Knoop, P. A. Andreasen, J. A. Andersen, S. Hansen, A. V. Laenkholm, A. C. Simonsen, J. Andersen, J. Overgaard, C. Rose

**Affiliations:** Department of Oncology, Odense University Hospital, Denmark.

## Abstract

The uPA-mediated pathway of plasminogen activation is central to cancer metastasis. Whether uPA and PAI-1 are related to local recurrence, metastatic spread or both is not clear. We present a retrospective study of 429 primary breast cancer patients with a median follow-up of 5.1 years, in which the levels of uPA and PAI-1 in tumour extracts were analysed by means of an enzyme-linked immunosorbent assay. The median values of uPA and PAI-1, which were used as cut-off points, were 4.5 and 11.1 ng mg(-1) protein respectively. The levels of uPA and PAI-1 were correlated with tumour size, degree of anaplasia, steroid receptor status and number of positive nodes. Patients with high content of either uPA or PAI-1 had increased risk of relapse and death. We demonstrated an independent ability of PAI-1 to predict distant metastasis (relative risk 1.7, confidence limits 1.22 and 2.46) and that neither uPA nor PAI-1 provided any information regarding local recurrence.


British Journal of Cancer (1998) 77(6), 932–940
© 1998 Cancer Research Campaign

### Prognostic significance of urokinase-type plasminogen activator and plasminogen activator inhibitor-1 in primary breast cancer

**A Knoop[1], PA Andreasen[2], JA Andersen[3], S Hansen[1], A-V Lænkholm[1], ACW Simonsen[2], J Andersen[4], J Overgaard[4] and C Rose[1]**

[1]Department of Oncology, Odense University Hospital, 5000 Odense C, Denmark; [2]Department of Molecular and Structural Biology, University of Aarhus, 8000 Aarhus C, Denmark; [3]Department of Pathology, Odense University Hospital, 5000 Odense C, Denmark; [4] The Danish Cancer Society, Clinical Department of Experimental Clinical Oncology, University of Aarhus, 8000 Aarhus C, Denmark

**Summary** The uPA-mediated pathway of plasminogen activation is central to cancer metastasis. Whether uPA and PAI-1 are related to local recurrence, metastatic spread or both is not clear. We present a retrospective study of 429 primary breast cancer patients with a median follow-up of 5.1 years, in which the levels of uPA and PAI-1 in tumour extracts were analysed by means of an enzyme-linked immunosorbent assay. The median values of uPA and PAI-1, which were used as cut-off points, were 4.5 and 11.1 ng $mg^{-1}$ protein respectively. The levels of uPA and PAI-1 were correlated with tumour size, degree of anaplasia, steroid receptor status and number of positive nodes. Patients with high content of either uPA or PAI-1 had increased risk of relapse and death. We demonstrated an independent ability of PAI-1 to predict distant metastasis (relative risk 1.7, confidence limits 1.22 and 2.46) and that neither uPA nor PAI-1 provided any information regarding local recurrence.

**Keywords:** urokinase; plasminogen; PAI-1; breast neoplasm mortality; prognosis

Cancer cells undergo the following steps during metastasis: detachment from the primary tumour; migration; invasion of the blood and lymphatic vessels; adhesion to and penetration of the endothelium, allowing colonization at distant sites (Liotta et al, 1991). Tumour progression and metastasis also involve various processes that may be called cancer cell-directed tissue remodelling. Examples are angiogenesis (Folkman, 1995) and desmoplasia (Dvorak et al, 1995).

Extracellular proteolysis has been implicated in cancer metastasis for many years, with the basic idea that release of proteolytic enzymes from a tumour leads to breakdown of basement membranes and extracellular matrix (ECM), thus allowing cancer cell invasion into the surrounding normal tissue. This is true for plasminogen activators and metalloproteinases (Danø et al, 1985; Liotta et al, 1991; Mignatti and Rifkin, 1993; Andreasen et al, 1997). There are two types of plasminogen activator, the urokinase-type uPA and the tissue-type tPA. Both are capable of catalysing the formation of the broad spectrum proteinase plasmin from the inactive zymogen plasminogen. There seems to be general agreement that uPA is most important for generation of plasmin in events involving degradation of ECM, while the primary role of tPA is to generate plasmin for thrombolysis. In relation to cancer metastasis, therefore, uPA is of main interest. There are two main types of inhibitors of plasminogen activator, PAI-1 and PAI-2. The urokinase receptor (uPAR) serves to localize plasminogen activation to cell surfaces (Danø et al, 1985; Andreasen et al, 1990, 1997; Mignatti and Rifkin, 1993).

*Received 18 April 1997*
*Revised 22 August 1997*
*Accepted 3 September 1997*

*Correspondence to:* A Knoop

Recent studies have shown that the levels of uPA and PAI-1 in malignant tumours vary considerably and are related to the prognosis of the patient (Andreasen et al, 1997). Whether uPA and PAI-1 are related to local recurrence, metastatic spread or both is not clear. None of the research groups has to our knowledge looked specifically into the levels of these proteins in relation to the site of relapse.

The purpose of the present retrospective study of 429 primary breast cancer patients was to relate tumour levels of uPA and PAI-1 to other prognostic factors and to the interval before local recurrence, first distant metastasis and death.

### MATERIALS AND METHODS

### Patients and tumours

From August 1984 to September 1989, 502 breast cancer patients underwent primary surgery at Odense University Hospital. All the patients came from an unscreened population. After routine diagnosis, residual tumour material was available in 447 patients. The material was stored at –80°C until the analysis for uPA and PAI-1. Information about tumour size was missing in 18 patients. The characteristics of the remaining 429 patients and their tumours are summarized in Table 1.

Most patients were entered in the Danish Breast Cancer Co-operative Group (DBCG) clinical trials (Andersen et al, 1981). Fifty-seven patients were treated with breast-conserving surgery and among those 48 had post-operative irradiation. Three hundred and seventy-five patients were treated with a simple mastectomy and of those 128 had post-operative irradiation. High-risk patients (N1, T3 or T4) were offered systemic adjuvant therapy. Eighty-five patients received a combination of cyclophosphamide, methotrexate and 5-fluorouracil (CMF); 109 patients received endocrine therapy, and 25 received a combination of CMF and tamoxifen. Two hundred and ten

**Table 1** Patient characteristics and correlation between uPA and other variables

| | *n* (%) | uPA median[a] | PAI-1 median[a] |
|---|---|---|---|
| Age (years) | | | |
| < 40 | 32 ( 7) | 4.4 | 10.5 |
| 40–49 | 76 (18) | 4.0 | 11.1 |
| 50–59 | 98 (23) | 4.3 | 10.6 |
| 60–69 | 100 (23) | 4.7 | 11.4 |
| ≥ 70 | 123 (29) | 4.8 | 11.2 |
| | | $P = 0.63$[b] | $P = 0.68$[b] |
| Median 61 | | | |
| Range 28–92 | | | |
| Menopausal status | | | |
| Pre | 137 (32) | 4.3 | 10.8 |
| Post | 292 (68) | 4.6 | 11.3 |
| | | $P = 0.54$[b] | $P = 0.41$[b] |
| Tumour size (mm) | | | |
| ≤ 20 | 156 (36) | 4.1 | 10.7 |
| 21–50 | 244 (57) | 5.0 | 12.1 |
| > 50 | 29 ( 7) | 4.1 | 10.1 |
| | | $P = 0.02$[b,c] | $P = 0.02$[b,c] |
| Median 25 | | | |
| Range 6–200 | | | |
| Differentiation grade | | | |
| I (Ductal) | 66 (15) | 4.1 | 10.3 |
| II (Ductal) | 156 (36) | 4.8 | 11.6 |
| III (Ductal) | 137 (32) | 5.2 | 12.9 |
| Other (not ductal) | 70 (16) | 2.3 | 7.5 |
| | | $P < 0.0001$[d] | $P < 0.0001$[d] |
| Receptor status | | | |
| Positive | 335 (78) | 4.3 | 11.0 |
| Negative | 94 (22) | 5.7 | 13.1 |
| | | $P = 0.008$[b] | $P = 0.004$[b] |
| No. of positive nodes | | | |
| 0 | 178 (41) | 4.1 | 9.9 |
| 1–3 | 144 (34) | 4.7 | 12.3 |
| ≥ 4 | 107 (25) | 5.2 | 13.7 |
| | | $P = 0.04$[b] | $P < 0.0001$[b] |
| Invasion of lymph node capsule | | | |
| No | 286 (67) | 4.4 | 10.3 |
| Yes | 143 (33) | 5.2 | 13.9 |
| | | $P = 0.27$[b] | $P = 0.0009$[b] |

[a]ng $mg^{-1}$ protein. [b]Kruskal–Wallis test. [c]Only significant difference between very small tumours and medium size tumours. [d]Only ductal carcinoma was included in the Kruskal–Wallis test.

patients received no systemic treatment, 178 patients were considered to be low risk (N0, T1–2) and 32 patients were either too old or were found to have some medical contraindications for systemic treatment. Patients were followed at regular intervals for a maximum of 10 years, and clinical data were obtained from patient records.

The end points for this study were:

- overall survival (OS, time from diagnosis to death from any cause);
- recurrence free (the probability of being without relapse; time from diagnosis to the appearance of new lesion(s) in patients with no previous evidence of disease, confirmed by physical examination, biopsies and/or other relevant diagnostic procedures) (death from cancer and death from other causes have been censored, because almost one-third of the patients were over 70 years of age, a fact that might blur the true picture of relapse);
- free of local recurrence (the probability of being without local recurrence, time to relapse within the local region);
- free of distant metastasis (the probability of being free of distant metastasis, time to metastasis outside the local region).

The result of the Cox regression analyses are summarized by the relative failure (failure was classified as death, any relapse, local relapse and distant metastasis).

The median time of observation was 61 months (1–128 months).

© Cancer Research Campaign 1998

### Histopathology

Tumour size (defined as the largest invasive tumour diameter), histological grade (Bloom and Richardson, 1957), number of axillary nodes and capsule invasion were estimated at routine pathological evaluation after primary surgery.

### Oestrogen and progesterone receptor assays

Oestrogen receptor (ER) and progesterone receptor (PgR) were analysed by the dextran-coated charcoal method in 371 patients (EORTC Breast Cancer Co-operative Group, 1980; Thorpe, 1987). The tumour was considered to be steroid receptor positive if the value of ER or PgR was greater than 10 fmol $mg^{-1}$ cytosol protein. The results were obtained from the DBCG's database. For the remaining 58 patients, the oestrogen receptor status was estimated semiquantitatively on paraffin-embedded tissue sections. The technique consisted of the Pathway HRP detection system (Pfeiffer et al, 1996). The antibody, clone 1D5 (Dako), was visualized by the substrate hydrogen peroxide and carbazol as the chromogen. We combined the results from both methods as numerous studies have shown concordance for ER-positive results in between 75% and 90% of the cases depending on the cut-off point used (Andersen et al, 1990; Esteban et al, 1994; De Mascarel et al, 1995; Alberts et al, 1996). The discordant results could be explained by a low level of ductal cell carcinoma (DCC) positivity or staining of non-malignant cells. The expected discordance in this study appears to be very low as all 58 patients investigated by immunohistochemistry had more than 50% of tumour cells positively stained.

### Proteins and antibodies for uPA and PAI-1

Human uPA was purchased from Serono, Aubonne, Switzerland. Human PAI-1, in the latent form, was prepared and converted to active and reactive centre-cleaved forms as described by Munch et al (1993). uPA/PAI-1 complex was prepared as described by Nykjær et al (1992). Human recombinant uPAR (Ploug et al, 1993) was provided by Dr N Behrendt, Finsen Laboratory, State University Hospital, Copenhagen, Denmark. The protein concentrations of the preparations were determined by amino acid analysis.

**Figure 1** Spearman's rank correlation between uPA and PAI-1: $r = 0.5675$, $P < 0.001$. Lines represent median values: uPA 4.5 ng $mg^{-1}$ protein, PAI-1 11.1 ng $mg^{-1}$ protein

Three clones of murine hybridomas, producing monoclonal anti-human uPA antibodies termed clones 2, 6 and 12 have been described previously (Grøndahl-Hansen et al, 1987; Pöllänen et al, 1987; Stephens et al, 1992). The epitopes for the three monoclonal antibodies are localized in the serine proteinase domain (antibody from clone 2) and the kringle domain (antibodies from clone 6 and 12) (Christensen et al, 1996). A rabbit polyclonal antibody was raised against uPA from Serono. A clone of murine hybridomas producing an anti-human PAI-1 antibody, clone 2, was the one described by Nielsen et al (1986*a*). The epitope for this monoclonal antibody is localized between amino acids 110 and 145 (Munch et al, 1991). A polyclonal rabbit antibody against PAI-1 was that described by Andreasen et al (1986). Horseradish peroxidase-conjugated swine antibodies directed against rabbit antibodies (P217) were purchased from Dako (Glostrup, Denmark). Other reagents were those previously described or were of the best grade commercially available (Munch et al, 1991; Nykjær et al, 1992; Christensen et al, 1996).

### ELISAs for uPA and PAI-1

Tissue for analysis was taken from –80°C and homogenized immediately in 0.1 M Tris, pH 8.1, 0.5% Triton X-100, 10 mM EDTA and 10 μg $ml^{-1}$ aprotinin (10 μl $mg^{-1}$ tissue) with an Ultraturrax with a S 25 N8G head (24 000 r.p.m.) at 4°C and centrifuged at 10 000 ***g*** for 10 min to remove cell debris and nuclei. The supernatants were analysed. The total protein concentration was determined using the Bradford method. For uPA ELISA, monoclonal anti-uPA IgG from hybridoma clones 2, 6 and 12 was used on the solid phase (2, 2 and 6 μg $ml^{-1}$ in the coating solution respectively). For PAI-1 ELISA, monoclonal anti-PAI-1 from hybridoma clone 2 was coated on the solid phase (8 μg $ml^{-1}$ in the coating solution). The second antibody layer consisted of polyclonal rabbit anti-uPA and rabbit anti-PAI-1 antibodies. As third layer, we used peroxidase-conjugated swine antibodies against rabbit antibodies for both ELISAs. The uPA and PAI-1 standards were the preparations described above. Other details of the ELISAs were as described by Nielsen et al (1986*b*).

The uPA ELISA gave the same signal per mol of uPA, uPA/PAI-1 complex, uPA/uPAR complex and PAI-1/uPA/uPAR complex (data not shown). The PAI-1 ELISA gave the same signal per mol of latent PAI-1, active PAI-1, reactive centre-cleaved PAI-1, uPA/PAI-1 complex and PAI-1/uPA/uPAR complex (data not shown) (Munch et al, 1991). The monoclonal anti-PAI-1 antibody from hybridoma clone 2 also reacted with vitronectin-bound PAI-1 (Andreasen, unpublished results). Internal standards added to tumour extracts were recovered with an efficiency above 90% (data not shown). In order to provide a further basis for comparison of our results with those obtained by other ELISAs, we analysed a reference sample designated '101094', kindly provided by Professor T Benraad, Department of Experimental and Chemical Endocrinology, Academic Hospital, University of Nijmegen, The Netherlands. The aliquots of freeze-dried powder provided (Grebenschikov et al, 1997) was reconstituted in 0.5 ml of a buffer of 0.01 M disodium hydrogen phosphate, pH 7.4, 0.15 M sodium chloride, 1% bovine serum albumin, 0.1% Tween 20. Our ELISAs gave a uPA concentration of 0.68 ± 0.06 ng $ml^{-1}$ (four determinations) and a PAI-1 concentration of 3.80 ± 0.17 ng $ml^{-1}$ (four determinations). For comparison, Grebenschikov et al (1997) estimated a uPA concentration of 0.90 ng $ml^{-1}$ and a PAI-1 concentration of 0.91 ng $ml^{-1}$. Thus, our ELISAs give a slightly lower

© Cancer Research Campaign 1998

value for uPA and a fourfold higher value for PAI-1 than that of Grebenschikov et al (1997).

### Statistical methods

Contingency tables were analysed using the $\chi^2$-test. Correlations between uPA and PAI-1 were calculated by Spearman's rank method. The correlation of uPA and PAI-1 with other prognostic factors was analysed using the Kruskal–Wallis test.

Probability rates of OS, being recurrence free and free of local recurrence and distant metastasis were calculated with life-table methods and compared using log-rank tests.

The Cox model of proportional hazards was used to study the independent effect on survival and recurrence of each variable. The classical prognostic factors (menopausal status, tumour size, grade, receptor status and number of positive nodes) and uPA and PAI-1 were included in the multivariate analysis. Two-sided *P*-values of less than 0.05 were considered to be significant.

### RESULTS

At the time of analysis, 201 of the 429 patients had relapsed (local recurrence 47, distant metastasis 126, both 28), 180 had died, 36 were alive with recurrence and 213 were alive with no recurrence.

**Table 2** Five-year survival

| Variable | *n* | Recurrence free % (s.e.) | Free of local recurrence % (s.e.) | Free of distant metastasis % (s.e.) | OS % (s.e.) |
|---|---|---|---|---|---|
| Menopausal status | | | | | |
| Pre | 137 | 62 (4) | 83 (3) | 67 (4) | 71 (4) |
| Post | 292 | 64 (3) | 90 (2) | 67 (3) | 61 (3) |
| | | $P = 0.53$ | $P = 0.06$ | $P = 0.96$ | **$P = 0.019$** |
| Tumour size (mm) | | | | | |
| ≤ 20 | 156 | 81 (3) | 93 (2) | 83 ( 3) | 79 (3) |
| 21–50 | 244 | 56 (3) | 85 (2) | 59 ( 3) | 56 (3) |
| > 50 | 29 | 33 (10) | 68 (9) | 45 (10) | 40 (9) |
| | | **$P < 0.0001$** | **$P = 0.001$** | **$P < 0.0001$** | **$P < 0.0001$** |
| Differentiation grade | | | | | |
| I (Ductal) | 66 | 88 (4) | 98 (2) | 90 (4) | 87 (4) |
| II (Ductal) | 156 | 61 (4) | 87 (3) | 65 (4) | 67 (4) |
| III (Ductal) | 137 | 52 (5) | 80 (4) | 57 (5) | 47 (4) |
| Other (not ductal) | 70 | 69 (6) | 88 (2) | 68 (6) | 66 (6) |
| | | **$P < 0.0001$** | **$P = 0.003$** | **$P = 0.0001$** | **$P < 0.0001$** |
| Receptor status | | | | | |
| Positive | 335 | 66 (3) | 89 (2) | 69 (3) | 65 (3) |
| Negative | 94 | 55 (5) | 81 (5) | 61 (6) | 57 (5) |
| | | $P = 0.07$ | $P = 0.89$ | $P = 0.16$ | $P = 0.12$ |
| No. of positive nodes | | | | | |
| 0 | 178 | 65 (4) | 88 (3) | 78 (3) | 76 (3) |
| 1–3 | 144 | 57 (4) | 93 (2) | 74 (4) | 68 (4) |
| ≥ 4 | 107 | 30 (5) | 76 (5) | 38 (5) | 38 (5) |
| | | **$P < 0.0001$** | **$P = 0.02$** | **$P < 0.0001$** | **$P < 0.0001$** |
| Invasion of capsule | | | | | |
| Yes | 143 | 40 (4) | 86 (3) | 50 (4) | 48 (4) |
| No | 286 | 61 (3) | 88 (2) | 75 (3) | 71 (3) |
| | | **$P < 0.0001$** | $P = 0.45$ | **$P < 0.0001$** | **$P < 0.0001$** |
| uPA quartile | | | | | |
| ≤ 2.3 | 108 | 71 (5) | 87 (4) | 75 (4) | 75 (5) |
| 2.4–4.5 | 111 | 71 (5) | 85 (4) | 76 (4) | 63 (5) |
| 4.6–7.0 | 104 | 58 (5) | 90 (3) | 60 (5) | 65 (5) |
| > 7.0 | 106 | 53 (5) | 85 (4) | 56 (5) | 53 (5) |
| | | **$P = 0.002$** | $P = 0.45$ | **$P = 0.001$** | **$P = 0.002$** |
| PAI-1 quartile | | | | | |
| ≤ 7.2 | 113 | 75 (4) | 88 (3) | 79 (4) | 78 (4) |
| 7.3–11.1 | 103 | 70 (5) | 85 (4) | 75 (4) | 68 (5) |
| 11.2–18.0 | 107 | 58 (5) | 87 (3) | 57 (5) | 52 (5) |
| > 18.0 | 106 | 52 (5) | 88 (4) | 54 (5) | 55 (5) |
| | | **$P < 0.0001$** | $P = 0.73$ | **$P < 0.0001$** | **$P < 0.0001$** |

*P*-values refer to result of the long-rank test. Numbers in bold type are significant.

© Cancer Research Campaign 1998

The distribution of uPA and PAI-1 concentrations in the 429 patients is shown in Table 1. Increased concentrations of uPA and PAI-1 were significantly correlated with increasing tumour size up to 50 mm, increasing grade, increasing number of positive nodes and negative steroid receptor status. PAI-1 was significantly related to invasion of the lymph node capsule. Neither the uPA nor PAI-1 was related to age or menopausal status.

High levels of uPA were weakly correlated with high levels of PAI-1 ($r = 0.5679$, $P < 0.001$) (Figure 1).

### Univariate prognostic analysis

The 5-year survival for classical prognostic factors and uPA and PAI-1 are listed in Table 2. Increasing size, grade, number of positive nodes and invasion of lymph node capsules were all indicators of decreased overall survival and recurrence-free probability.

The table shows that breast cancer patients with high content of either uPA or PAI-1 in their primary tumours had an increased risk of relapse and death. The probability of being without recurrence after 5 years with a uPA or PAI-1 value below median was 71% and 73%, respectively, and above median 56% and 54%. The same picture was seen for overall survival. When specifically studying the site of relapse, local or distant, uPA and PAI-1 were prognostic only for the end point free of distant metastasis. The probability of being without distant metastasis after 5 years with a uPA or PAI-1 value below median was 76% and 78%, respectively, and above median 58% and 56%. By contrast, the probability of local control after 5 years was approximately 88%, regardless of the uPA or PAI-1 value. We found no difference in locoregional recurrence after stratifying the patients into type of primary local treatment (data not shown).

In Table 2, the 5-year probabilities for the different end points, according to uPA and PAI-1, are listed for the quartiles. Looking at

**Figure 2** Kaplan–Meier curves, with 5-year probabilities for different end points. Dotted lines present uPA or PAI-1 values below median. Solid lines present uPA or PAI-1 above median. *P*-values from log-rank test

© Cancer Research Campaign 1998

**Table 3** Multivariate analysis

| Variable $n = 429$ | Death | | | Any failure (local or distant) | | | Distant metastasis | | |
|---|---|---|---|---|---|---|---|---|---|
| | *P*-value | RR | CL 95% | *P*-value | RR | CL 95% | *P*-value | RR | CL 95% |
| Menopausal status[a] | 0.0013 | 1.7 | 1.24–2.40 | 0.96 | | | 0.55 | | |
| Size[b] | 0.0003 | 1.6 | 1.25–2.13 | 0.0001 | 1.8 | 1.33–2.38 | 0.0009 | 1.7 | 1.22–2.23 |
| Grade[c] | 0.0001 | 1.6 | 1.28–2.08 | 0.0155 | 1.4 | 1.06–1.78 | 0.0245 | 1.4 | 1.04–1.77 |
| Receptor status[d] | 0.5 | | | 0.82 | | | 0.51 | | |
| Lymph node status[e] | <0.0001 | 1.6 | 1.36–1.99 | <0.0001 | 1.7 | 1.4–2.13 | <0.0001 | 1.7 | 1.39–2.10 |
| uPA median[f] | 0.6 | | | 0.17 | | | 0.19 | | |
| PAI-1 median[g] | 0.0014 | 1.6 | 1.20–2.22 | 0.005 | 1.6 | 1.15–2.25 | 0.0015 | 1.7 | 1.22–2.46 |

[a]Score: 1, premenopausal; 2, post-menopausal. [b]Score: 1, ≤ 20 mm; 2, 21–50 mm; 3, > 50 mm. [c]Score: 1, grade I; 3, grade III; 2, all others (grade II and other than ductal carcinoma). [d]Score: 1, positive for either oestrogen and/or progesterone; 2, negative for both. [e]Score: 1, no positive nodes; 2, one to three positive nodes; 3, ≥ four positive nodes. [f]Score: 1, below median (4.45 ng $mg^{-1}$ protein); 2, above median. [g]Score: 1, below median (11.1 ng $mg^{-1}$ protein); 2, above median

**Table 4** uPA and relation to different end points in multivariate analysis (No subgroup analysis included)

| References | *n*[a] | Median follow-up (months) | N+ (%) | ER+ (%) | Post-menopausal (%) | Systemic adjuvant therapy (%) | PAI-1 in analysis | Independent prognostic relation to end point in multivariate analysis: Any failure (local + distant) | DM (distant metastasis) | Death |
|---|---|---|---|---|---|---|---|---|---|---|
| Duffy et al (1990,1994) | 166 | 68 | 45 | 58 | 59 | 69 | No | Yes | – | Yes |
| Jänicke et al (1990,1991) | 113 | 26 | 54 | 73 | 60 | – | Yes | Yes | – | Yes |
| Jänicke et al (1993) | 229 | 30 | 64 | 56 | 69 | min.64 | Yes | Yes | – | Yes |
| Foekens et al (1994*a*) | 618 | 48 | 58 | 74 | 62 | 25 | Yes | Yes | – | No |
| Foekens et al (1994*b*) | 587 | 51 | 56 | – | – | 22 | Yes | – | Yes | – |
| Spyratos et al (1992) | 319 | 72 | 53 | 70 | 55 | 37 | No | No | Yes | – |
| Bouchet et al (1994) | 314 | 84 | 53 | 70 | 55 | 37 | Yes | No | No | – |
| Present study | 429 | 61 | 59 | 77 | 68 | 51 | Yes | No | No | No |

[a]Maximum number included in the multivariate analysis.

**Table 5** PAI-1 and relation to different end points in multivariate analysis (No subgroup analysis included)

| References | *n*[a] | Median follow-up (months) | N+ (%) | ER+ (%) | Post-menopausal (%) | Systemic adjuvant therapy (%) | uPA in analysis | Independent prognostic relation to end point in multivariate analysis: Any failure (local + distant) | DM (distant metastasis) | Death |
|---|---|---|---|---|---|---|---|---|---|---|
| Jänicke et al (1990, 1991) | 113 | 26 | 54 | 73 | 60 | – | Yes | Yes | – | No |
| Jänicke et al (1993) | 229 | 30 | 64 | 56 | 69 | min.64 | Yes | No | – | No |
| Foekens et al (1994*a*) | 618 | 48 | 58 | 74 | 62 | 25 | Yes | Yes | – | Yes |
| Foekens et al (1994*b*) | 587 | 51 | 56 | – | – | 22 | Yes | – | Yes | – |
| Bouchet et al (1994) | 314 | 84 | 53 | 70 | 55 | 37 | Yes | Yes | Yes | – |
| Present study | 429 | 61 | 59 | 77 | 68 | 51 | Yes | Yes | Yes | Yes |

[a]Maximum number included in the multivariate analysis.

the different probabilities, it seems reasonable to divide the patients into low- and high-risk groups according to their median values. The Kaplan–Meier curves for all end points according to uPA and PAI-1 are illustrated in Figure 2.

In order to find out whether uPA and/or PAI-1 gave any more information than the classical factors in the node-negative group, we looked into differently constructed low-risk subgroups: a pure node-negative group ($n = 178$) and a node-negative, receptor-positive group ($n = 136$). We investigated whether uPA above median, PAI-1 above median, high uPA and/or high PAI-1 were able to split these low-risk groups into two significantly different 5-year probabilities for all end points. But neither uPA nor PAI-1, alone or

© Cancer Research Campaign 1998

in combination, gave any additional information, although high uPA and/or PAI-1 showed decreased but non-significant survival for all end points, except for local control (data not shown). This conclusion has of course to be taken with great caution, because the groups are very small.

### Multivariate prognostic analysis

Table 3 shows the results of the Cox multivariate regression analysis, which used all the classical variables (menopausal status, size, grade, receptor status, number of positive nodes) and uPA and PAI-1 with cut-off points defined by their medians. The non-ductal carcinomas were scored together with ductal grade II carcinoma because they had approximately the same survival.

The analysis showed that size, positive nodes, grade, post-menopausal status and high PAI-1 were independent predictors of OS, with a relative risk (RR) of 1.6–1.7. Size, positive nodes, grade and high PAI-1 were independent predictors of any relapse, distant and local, with an RR between 1.4 and 1.8. When the site of relapse was studied, size, positive nodes, grade and high PAI-1 were independent predictors of distant metastasis (RR 1.4–1.7) Only increasing size (RR 1.9, confidence limit (CL) 1.18–3.09) and grade (RR 1.6, CL 1.03–2.49) retained independent prognostic information for the end point local relapse (data not shown).

Our results show that, when looking at the specific site of relapse according to the plasminogen activator system, only PAI-1, and only with respect to predicting distant metastasis, has independent information. The uPA retained its independent prognostic significance for none of the end points.

### DISCUSSION

Duffy et al (1988) were the first to report a shorter disease-free survival of breast cancer patients with higher levels of tumour uPA enzyme activity, and Duffy et al (1990) reported a shorter disease-free survival for patients with higher levels of tumour uPA antigen. Jänicke et al (1991) first reported a correlation between tumour PAI-1 antigen levels and poor prognosis of breast cancer. Since then, a number of other studies have confirmed that the uPA and PAI-1 tumour levels are correlated with poor prognosis in several cancer types (Duffy, 1996; Andreasen et al, 1997). Results of such studies, with more than 100 patients, are summarized in Tables 4 and 5.

Our breast cancer patient material is the second largest analysed for uPA and PAI-1. Our results show the same general trend as the previous studies, i.e. the correlation between uPA and PAI-1 antigen levels and poor prognosis. However, our study, which revealed some important previously unknown results, differs in several respects from those of others.

In our study, high levels of uPA and PAI-1 were correlated with increasing number of involved lymph nodes, grade, tumour size and low content of ER or PgR. This contrasts with the majority of the larger published studies, in which this correlation was not found for uPA (Foucré et al, 1991; Jänicke et al, 1991, 1993; Foekens et al, 1992; Spyratos et al, 1992; Grøndahl–Hansen et al, 1993; Bianchi et al, 1994; Bouchet et al, 1994; Göhring et al, 1995) and only partly for PAI-1 (Foucré et al, 1991; Jänicke et al, 1991, 1993; Grøndahl–Hansen et al, 1993; Foekens et al, 1994*a*). In agreement with all earlier published studies, we found a correlation between uPA and PAI-1 (Jänicke et al, 1990, 1991, 1993; Bouchet et al, 1994; Foekens et al, 1994*a* and *b*).

In contrast to our results, Table 4 shows uPA as an independent prognostic factor for overall survival in three out of four studies, and for recurrence-free or disease-free survival in five out of seven studies. Although the patients of Spyratos et al (1992) and Bouchet et al (1994) seem similar, the first study included cathepsin D and not PAI-1, while the second study did the opposite. This is no doubt the main explanation for the different results regarding metastasis-free survival (MFS). Foekens et al (1994*b*) included both cathepsin D and PAI-1 and found uPA to be an independent prognostic factor for metastasis-free survival (MFS).

The picture for PAI-1 is more consistent because, in all studies (Jänicke et al, 1990, 1991; Bouchet et al, 1994; Foekens et al, 1994*a* and *b*) but one, PAI-1 retains its independent prognostic significance for recurrence. The one study, which in the overall population was non-significant (Jänicke et al, 1993), showed PAI-1 as an independent prognosticator in a subgroup analysis of the node-negative group. We could not confirm this, even though our node-negative group comprised more patients and had a longer follow-up period.

The new aspect of our study was that neither uPA nor PAI-1 predicted local control and that in the multivariate analysis the prediction of distant relapse was related to PAI-1 but not to uPA. The last was in agreement with the study from Bouchet et al (1994) who in the multivariate analysis regarding distant relapse found that PAI-1 but not uPA retained its prognostic power. In contrast to this, Foekens et al (1994*b*) found both uPA and PAI-1 to be independent predictors of distant metastasis.

There are problems in comparing the different studies because the length of follow-up, the distribution of lymph node-positive patients, the number of patients receiving adjuvant therapy and so forth differ between the studies, as illustrated. Furthermore, the multivariate analysis did not include the same variables, and all the studies, except in part the study from Foekens et al (1994*b*) and ours, used optimal cut-off points as the cut-off values in the model. This may be the main reason for the different results obtained in the different studies.

The median uPA and PAI-1 values that we found in the tumour extract are higher than those previously reported. We have used Triton X-100 extracts of tumours, while several researchers use so-called 'cytosols', originally prepared and used for steroid receptor analysis. In our hands, the Triton X-100 extraction yields 19-fold higher values for uPA and sixfold higher values for PAI-1 (data not shown). Moreover, we have ensured that the antibody combination used in the ELISA is not only specific but also gives the same signal with all forms of the antigens that are conceivable at the moment.

It has been claimed that uPA in detergent extracts is a stronger prognostic parameter than cytolic uPA, and that this could explain the fact that uPA did not retain the prognostic power in the multivariate analysis together with PAI-1 when cytolic uPA was used (Grøndahl-Hansen et al, 1993). This could not be confirmed in our study.

The results from this relatively large patient group showed the significant relationship between uPA and PAI-1 and most known prognostic factors, and confirmed other studies showing uPA and PAI-1 as giving prognostic information, although uPA did not emerge as an independent factor. The major new findings were the absent prognostic information of uPA and PAI-1 with respect to local control and the ability of PAI-1 to independently predict distant metastasis.

The correlation between poor prognosis and a high level of uPA is in agreement with the basic idea that uPA is necessary for cancer

 © Cancer Research Campaign 1998

cell invasion. The finding that PAI-1 is a strong prognostic marker in cancer emphasizes our present lack of in-depth understanding of its functions in cancer. Although many new findings concerning the cellular actions of PAI-1 have been reported recently, the cellular mechanisms behind the correlation in the case of PAI-1 is not completely understood (Andreasen et al, 1997).

### ACKNOWLEDGEMENTS

This study was supported by grants from the Danish Cancer Society. The authors wish to thank Dr Susan M Thorpe for providing us with her results from the analysis of the oestrogen and progesterone receptors. Drs T Benraad, N Grebenschikov and H De Witte are thanked for helpful suggestions.

### REFERENCES

Alberts SR, Ingle JN, Roche PR, Cha SS, Wold LE, Farr GH Jr, Krook JE and Wieand HS (1996) Comparison of estrogen receptor determinations by a biochemical ligand-binding assay and immunohistochemical staining with monoclonal antibody ER1D5 in females with lymph node positive breast carcinoma entered on two prospective clinical trials. *Cancer* **78**: 764–772

Andersen KW, Mouridsen HT, Castberg T, Fischerman K, Andersen J, Hou-Jensen K, Brincker H, Johansen H, Henriksen E, Roth M and Rossing N (1981) Organization of the Danish adjuvant trials in breast cancer. *Danish Med Bull* **28**: 102–106

Andersen J, Thorpe SM, King WJ, Rose C, Christensen I, Rasmussen BB and Poulsen HS (1990) The prognostic value of immunohistochemical estrogen receptor analysis in paraffin-embedded and frozen sections versus that of steroid-binding assays. *Eur J Cancer* **26**: 442–449

Andreasen PA, Nielsen LS, Kristensen P, Grøndahl-Hansen J, Skriver L and Danø K (1986) Plasminogen activator inhibitor from human fibrosarcoma cells binds urokinase-type plasminogen activator but not its proenzyme. *J Biol Chem* **261**: 7644–7657

Andreasen PA, Georg B, Lund LR, Riccio A and Stacey SN (1990) Plasminogen activator inhibitors: hormonally regulated serpins. *Mol Cell Endocrinol* **68**: 1–19

Andreasen PA, Kjøller L, Christensen L and Duffy MJ (1997) The urokinase-type plasminogen activator system in cancer metastasis. A review. *Int J Cancer* **71**: 1–22

Bianchi E, Cohen RL, Thor AT, Todd RF 3rd, Mizukami IF, Lawrence DA, Ljung BM, Shuman MA and Smith HS (1994) The urokinase receptor is expressed in invasive breast cancer but not in normal breast tissue. *Cancer Res* **54**: 861–866

Bloom HJG and Richardson WW (1957) Histological grading and prognosis in breast cancer. A study of 1409 cases of which 359 have been followed for 15 years. *Br J Cancer* **11**: 359–377

Bouchet C, Spyratos F, Martin PM, Hacene K, Gentile A and Oglobine J (1994) Prognostic value of urokinase-type plasminogen activator (uPA) and plasminogen activator inhibitors PAI-1 and PAI-2 in breast carcinomas. *Br J Cancer* **69**: 398–405

Christensen L, Simonsen ACW, Heegaard CW, Moestrup SK, Andersen JA and Andreasen PA (1996) Immunohistochemical localization of urokinase-type plasminogen activator in human breast carcinomas. *Int J Cancer* **66**: 441–452

Danø K, Andreasen PA, Grøndahl-Hansen J, Kristensen P, Nielsen LS and Skriver L (1985) Plasminogen activators, tissue degradation, and cancer. *Adv Cancer Res* **44**: 139–266

De Mascarel I, Soubeyran I, MacGrogan G, Wafflart J, Bonichon F, Durand M, Avril A, Mauriac L, Trojani M and Coindre JM (1997) Immunohistochemical analysis of estrogen receptors in 938 breast carcinomas. Concordance with biochemical assay and prognostic significance. *Appl Immunohistochem* **3**: 222–231

Duffy MJ (1996) Proteases as prognostic markers in cancer. *Clin Cancer Res* **2**: 613–618

Duffy MJ, O'Grady P, Devaney D, O'Siorain L, Fennelly JJ and Lijnen HJ (1988) Urokinase plasminogen activator, a marker for aggressive breast carcinomas. Preliminary report. *Cancer* **62**: 531–533

Duffy MJ, Reilly D, O'Sullivan C, O'Higgins N, Fennelly JJ and Andreasen P (1990) Urokinase-plasminogen activator, a new and independent prognostic marker in breast cancer. *Cancer Res* **50**: 6827–6829

Duffy MJ, Reilly D, McDermott E, O'Higgins N, Fennelly JJ and Andreasen PA (1994) Urokinase plasminogen activator as a prognostic marker in different subgroups of patients with breast cancer. *Cancer* **74**: 2276–2280

Dvorak HF, Brown LF, Detmar M and Dvorak AM (1995) Vascular permeability factor/vascular endothelial growth factor, microvascular hyperpermeability and angiogenesis. *Am J Pathol* **146**: 1029–1039

EORTC Breast Cancer Co-operative Group (1980) Revision of the standards for assessment of hormone receptors in human breast cancer. *Eur J Cancer* **16**: 1513–1515

Esteban JM, Ahn C, Battifora H and Felder B (1994) Quantitative immunohistochemical assay for hormonal receptors: technical aspects and biological significance. *J Cell Biochem Suppl* **19**: 138–145

Fernö M, Bendahl PO, Borg A, Brundell J, Hirschberg L, Olsson H and Killander D (1996) Urokinase plasminogen activator, a strong independent prognostic factor in breast cancer, analysed in steroid receptor cytosols with a luminometric immunoassay. *Eur J Cancer* **32A**: 793–801

Foekens JA, Schmitt M, van Putten WL, Peters HA, Bontenbal M, Jänicke F and Klijn JG (1992) Prognostic value of urokinase-type plasminogen activator in 671 primary breast cancer patients. *Cancer Res* **52**: 6101–6105

Foekens JA, Schmitt M, van Putten WL, Peters HA, Kramer MD, Jänicke F and Klijn JG (1994*a*) Plasminogen activator inhibitor-1 and prognosis in primary breast cancer. *J Clin Oncol* **12**: 1648–1658

Foekens JA, Schmitt M, Peters HA, Look MP, van Putten WL, Kramer MD, Jänicke F and Klijn JG (1994*b*) Association of PAI-1 with metastasis-free survival in breast cancer: comparison with ER, PgR, PS2, cathepsin D and uPA. In *Prospectives in Diagnosis and Treatment of Breast Cancer*, Schmidt M (ed), pp. 197–205. Excerpta Medica: Amsterdam

Folkman J (1995) Angiogenesis in cancer, vascular, rheumatoid and other diseases. *Nature Med* **1**: 27–31

Foucré D, Bouchet C, Hacene K, Pourreau Schneider N, Gentile A, Martin PM, Desplaces A and Oglobine J (1991) Relationship between cathepsin D, urokinase, and plasminogen activator inhibitors in malignant vs benign breast tumours. *Br J Cancer* **64**: 926–932

Göhring UJ, Scharl A, Thelen U, Ahr A and Titius BR (1995) Prognostic value of immunohistochemical determination of urokinase plasminogen activator in primary breast cancer. *Pathologe* **14**: 398–403

Grebenschikov N, Geurts-Moespot A, De Witte H, Heuvel J, Leake R, Sweep F and Benraad T (1997) A sensitive and robust assay for urokinase and tissue type plasminogen activators (uPA and tPA) and their inhibitor type I (PAI-1) in breast tumor cytosols. *Int J Biol Markers* **12**: 6–14

Grøndahl-Hansen J, Ralfkiaer E, Nielsen LS, Kristensen P, Frentz G and Danø K (1987) Immunohistochemical localization of urokinase- and tissue-type plasminogen activators in psoriatic skin. *J Invest Dermatol* **88**: 28–32

Grøndahl-Hansen J, Christensen IJ, Rosenquist C, Brunner N, Mouridsen HT, Danø K and Blichert Toft M (1993) High levels of urokinase-type plasminogen activator and its inhibitor PAI-1 in cytosolic extracts of breast carcinomas are associated with poor prognosis. *Cancer Res* **53**: 2513–2521

Jänicke F, Schmitt M, Hafter R, Hollrieder A, Ulm K, Grossner W and Graeff H (1990) Urokinase-type plasminogen activator (u-PA) antigen is a predictor of early relapse in breast cancer. *Fibrinolysis* **4**: 69–78

Jänicke F, Schmitt M and Graeff H (1991) Clinical relevance of the urokinase-type and tissue-type plasminogen activators and of their type 1 inhibitor in breast cancer. *Semin Thromb Hemost* **17**: 303–312

Jänicke F, Schmitt M, Pache L, Ulm K, Harbeck N, Hofler H and Graeff H (1993) Urokinase (uPA) and its inhibitor PAI-1 are strong and independent prognostic factors in node-negative breast cancer. *Breast Cancer Res Treat* **24**: 195–208

Liotta LA, Steeg PS and Stetler-Stevenson WG (1991) Cancer metastasis and angiogenesis: an imbalance of positive and negative regulation. *Cell* **64**: 327–336

Mignatti P and Rifkin DB (1993) Biology and biochemistry of proteinases in tumor invasion. *Physiol Rev* **73**: 161–195

Munch M, Heegaard C, Jensen PH and Andreasen PA (1991) Type-1 inhibitor of plasminogen activators. Distinction between latent, activated and reactive centre-cleaved forms with thermal stability and monoclonal antibodies. *FEBS Lett* **295**: 102–106

Munch M, Heegaard CW and Andreasen PA (1993) Interconversions between active, inert and substrate forms of denatured/refolded type-1 plasminogen activator inhibitor. *Biochim Biophys Acta* **1202**: 29–37

Nielsen LS, Andreasen PA, Grøndahl-Hansen J, Huang JY, Kristensen P and Danø K (1986*a*) Monoclonal antibodies to human 54 000 molecular weight plasminogen activator inhibitor from fibrosarcoma cells – inhibitor neutralization and one-step affinity purification. *Thromb Haemost* **55**: 206–212

© Cancer Research Campaign 1998 

Nielsen LS, Grøndahl-Hansen J, Andreasen PA, Skriver L, Zeuthen J and Danø K (1986*b*) Enzyme-linked immunosorbent assay for human urokinase-type plasminogen activator and its proenzyme using a combination of monoclonal and polyclonal antibodies. *J Immunoassay* **7**: 209–228

Nykjær A, Petersen CM, Møller B, Jensen PH, Moestrup SK, Holtet TL, Etzerodt M, Thogersen HC, Munch M and Andreasen PA (1992) Purified alpha 2-macroglobulin receptor/LDL receptor-related protein binds urokinase plasminogen activator inhibitor type-1 complex. Evidence tha the alpha 2-macroglobulin receptor mediates cellular degradation of urokinase receptor-bound complexes. *J Biol Chem* **267**: 14543–14546

Pfeiffer P, Grabau DA, Nielsen O and Clausen PP (1996) Immunohistochemical bulk staining of slides using a rack peroxidase-labelled streptavidin-biotin technique. *Appl Immunohistochem* **4**: 135–138

Ploug M, Kjalke M, Rønne E, Weidle U, Hoyer Hansen G and Danø K (1993) Localization of the disulfide bonds in the NH2-terminal domain of the cellular receptor for human urokinase-type plasminogen activator. A domain structure belonging to a novel superfamily of glycolipid-anchored membrane proteins. *J Biol Chem* **268**: 17539–17546

Pöllänen J, Saksela O, Salonen EM, Andreasen P, Nielsen L, Danø K and Vaheri A (1987) Distinct localizations of urokinase-type plasminogen activator and its type 1 inhibitor under cultured human fibroblasts and sarcoma cells. *J Cell Biol* **104**: 1085–1096

Spyratos F, Martin PM, Hacene K, Romain S, Andrieu C, Ferrero Pous M, Deytieux S, Le Doussal V, Tubiana Hulin M and Brunet M (1992) Multiparametric prognostic evaluation of biological factors in primary breast cancer. *J Natl Cancer Inst* **84**: 1266–1272

Stephens RW, Bokman AM, Myohanen HT, Reisberg T, Tapiovaara H, Pedersen N, Grøndahl Hansen J, Llinas M and Vaheri A (1992) Heparin binding to the urokinase kringle domain. *Biochemistry* **31**: 7572–7579

Thorpe SM (1987) Steroid receptors in breast cancer: sources of inter-laboratory variation in dextran-charcoal assays. *Breast Cancer Res Treat* **11**: 175–189

*© Cancer Research Campaign 1998*